# The role and mechanism of TXNDC5 in diseases

**DOI:** 10.1186/s40001-022-00770-4

**Published:** 2022-08-08

**Authors:** Xueling Wang, Haoran Li, Xiaotian Chang

**Affiliations:** 1 Medical Research Center of The Affiliated Hospital of Qingdao University, No 1677 Wutaishan Road, Huangdao District, Qingdao, China; 2 Department of Hepatobiliary and Pancreatic Surgery, Affiliated Hospital of Qingdao University, No 16 Jiangsu Road, Qingdao, China

**Keywords:** Thioredoxin domain-containing protein 5 (TXNDC5), Cancer, Rheumatoid arthritis (RA), Diseases

## Abstract

Thioredoxin domain-containing protein 5 (TXNDC5) is a member of the protein disulfide isomerase (PDI) family. It can promote the formation and rearrangement of disulfide bonds, ensuring proper protein folding. TXNDC5 has three Trx-like domains, which can act independently to introduce disulfide bonds rapidly and disorderly. TXNDC5 is abnormally expressed in various diseases, such as cancer, rheumatoid arthritis (RA), etc. It can protect cells from oxidative stress, promote cell proliferation, inhibit apoptosis and promote the progression of disease. Aberrant expression of TXNDC5 in different diseases suggests its role in disease diagnosis. In addition, targeting TXNDC5 in the treatment of diseases has shown promising application prospects. This article reviews the structure and function of TXNDC5 as well as its role and mechanism in cancer, RA and other diseases.

## Background

Thioredoxin domain-containing protein 5 (TXNDC5) is a member of the protein disulfide isomerase (PDI) family, and it is also known as ERp46, PDIA15, HCC-2 or Endo-PDI. TXNDC5 is a protein with a molecular weight of 48 kDa [1]. It was first found to be highly expressed in endothelial cells, liver cells and hypoxic tissues, such as cancer endothelial cells and atherosclerotic plaques [1, 2]. The TXNDC5 protein is mainly expressed in the endoplasmic reticulum (ER), and it is also expressed in the cytoplasm and envelope [3]. The TXNDC5 gene is located on chromosome 6p24.3 and encodes six indirect variants, two of which (TXNDC5-001 and TXNDC5-003) can be translated into proteins. TXNDC5-001 is a full-length protein containing 432 amino acids, and TXNDC5-003 isoform consists of 389 amino acids [4]. The current research on TXNDC5 always refers to the full-length one. In addition, compared with archetypal PDI family members, TXNDC5 has unique structure and function. Since the discovery of TXNDC5, many studies have found its abnormal expression in various diseases. Single nucleotide polymorphisms (SNPs) in the TXNDC5 gene are associated with the risk of various diseases (Table 1). In addition, TXNDC5 plays an important role in cell proliferation, apoptosis, migration and antioxidative stress. Besides, it has shown a promising role in the diagnosis and treatment of diseases. The role of TXNDC5 in diseases has been extensively studied in recent years. Among them, cancers and rheumatoid arthritis (RA) are most widely studied. In this paper, we summarize the structural and functional characteristics of TXNDC5 and its role in cancer, RA and other diseases.

**Table 1:** TXNDC5 SNP-related diseases

Diseases	SNP	Gene symbol	Location	Ref
Oesophageal cancer	rs1632346 rs9505309 rs2815128 rs2815142	TXNDC5/MUTED MUTED/TXNDC5 MUTED MUTED	INTERGENIC INTERGENIC INTRON MUTED	[21]
Liver cancer	rs13210097 rs11754300 rs9392182 rs2815128	TXNDC5/MUTED TXNDC5/MUTED MUTED/TXNDC5 MUTED	INTERGENIC INTERGENIC INTERGENIC INTRON	[21]
Cervical cancer	rs408014 rs7771314 rs9505298	TXNDC5 TXNDC5 BMP6/TXNDC5	INTRON INTRON COMPLEX	[21, 26]
Hepatocellular carcinoma	rs1225944 rs1225943	TXNDC5 TXNDC5	INTRON INTRON	[27]
Rheumatoid arthritis	rs9505298 rs41302895 rs1225936 rs1225938 rs372578 rs443861 rs408014 rs9392189 rs2743992	BMP6/TXNDC5 TXNDC5/BMP6 TXNDC5 TXNDC5 TXNDC5 TXNDC5 TXNDC5 MUTED MUTED	COMPLEX COMPLEX INTRON INTRON INTRON INTRON INTRON INTRON INTRON	[47]
Nonsegmental vitiligo	rs1043784 rs7764128 rs8643	TXNDC5 TXNDC5 TXNDC5	Exon Exon Exon	[52]
Schizophrenia	rs13873 rs1225934–rs13873	TXNDC5 BMP6–TXNDC5	INTRON -	[53]

### TXNDC5 structure

**Fig. 1:**
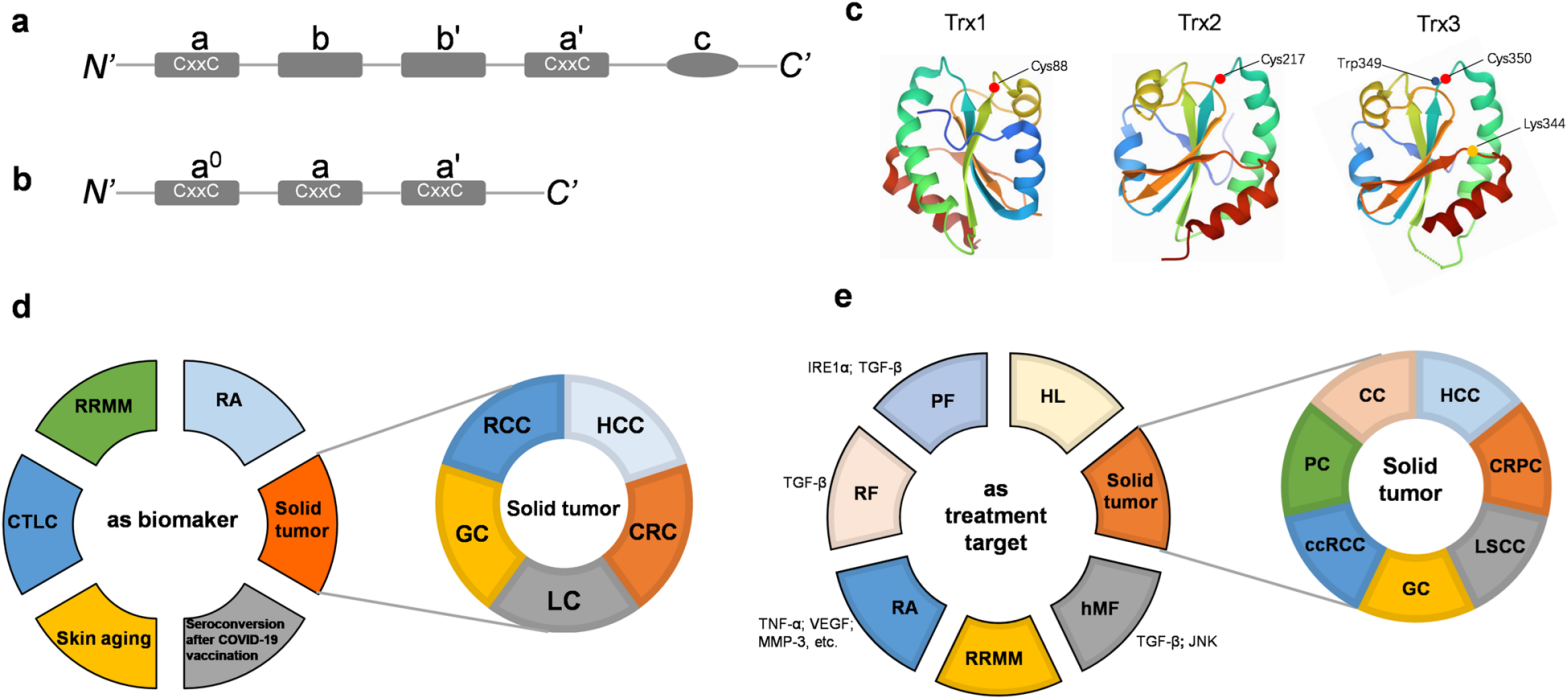
Scheme showing archetypal PDI and TXNDC5 domains and the role of TXNDC5 in diseases. Compared with the archetypal PDI domain (**a**), TXNDC5 has three a domains and no b domains (**b**); **c**: three TrX-like domains of TXNDC5 and their substrate binding sites; TXNDC5can be used as disease diagnostic biomarker (**d**) and treatment target (**e**). The surrounding text indicates the acting substrate or signal pathway. *CC* cervical cancer, *ccRCC* clear cell renal cell carcinoma, CRC colorectal cancer; *CRPC* castration-resistant prostate cancer, *CTLC* cutaneous T-cell lymphoma, *GC* gastric cancer, *HCC* hepatocellular carcinoma, *HL* hepatic Lipidosis, *hMF* human myocardial fibrosis, *LC* lung cancer, *PF* pulmonary fibrosis, *LSCC* laryngeal squamous cell carcinoma, *PC* pancreatic cancer, *RA* rheumatoid arthritis, *RCC* renal cell carcinoma, *RF* renal fibrosis, *RRMM* refractory/relapsed multiple myeloma;

TXNDC5 is a member of the PDI family. PDI consists of four Trx-like domains (**a**, **b**, **bʹ**, **aʹ**) and a C-terminal domain (**c**) (Fig. 1a). The **a** and **aʹ** domains have redox activity due to the CxxC motif, and it can also catalyse the formation of natural disulfide bonds [5]. The **b** and **b'** domains have no CxxC motif and, therefore, no oxidoreductase activity. However, the **bʹ** domain is mainly responsible for providing substrate binding sites [6, 7]. In addition, it binds to unfolded or partially folded protein substrates [8]. Unlike archetypal PDI, TXNDC5 contains three Trx-like domains (**a**^**0**^, **a** and **aʹ**) [1, 9] (Fig. 1b) that all have redox active sites. In addition, the three different domains are connected by approximately 20 amino acid residues [10]. Three Trx-like domains of TXNDC5 can act independently to accelerate protein folding, while the Trx-like domains of PDI require synergy [10]. In the absence of the **b** domain, the three catalytic domains of TXNDC5 were found to bind peptides containing aromatic or alkaline residues [11]. Besides, cysteine sites of Trx-like domains Cys88, 217 and 350 are considered with supposed protein binding function [10] (Fig. 1c). The interaction observed in TXNDC5 crystals suggests that the third catalytic domain of TXNDC5 may bind to the substrate through small hydrophobic pockets on the surface of the Trx-like domain or by exposing the Trp349 residues [12]. A specific structural feature of TXNDC5 is the presence of a lysine residue (Lys344) near the second cysteine in the CxxC motif [12]. The lysine plays a role in the catalytic reaction by forming a salt bridge with glutamate 378 (Glu378) on the adjacent β chain. However, in other archetypal members of PDIs, glutamate plays a catalytic role by accepting the proton of the second cysteine [12]. Despoina et al. found that the reactivity of Trx-fold proteins is not determined solely by their CxxC motif. The cysteine pKa values, which determine reactivity, are fine-tuned by interactions involving several residues distant in sequence but spatially close to the CxxC motif [13]. In PDIA1, arginine residue Arg415 can participate in the catalytic reaction by regulating the pKa value of the second cysteine in the CxxC motif [14]. In the third Trx-like domain of TXNDC5, it was observed that Arg415 did not insert into the hydrophobic nucleus but bonded to the carbonyl oxygen of Pro397 by hydrogen bonds, so it couldn’t regulate the cysteine PKa value [11].

### TXNDC5 function

Similar to most members of the PDI family, TXNDC5 can catalyse the formation and rearrangement of disulfide bonds, thereby promoting the correct folding of proteins. In addition, the CxxC motif plays a key role in this process. Properly formed disulfide bonds can enhance the stability of proteins and protect them from damage [15, 16]. Both PDI and TXNDC5 can catalyse the formation of regulatory disulfide in endoplasmic reticulum oxidoreductase 1α (Ero1α), thereby preventing excessive oxidative stress caused by excessive H_2_O_2_ production. Ero1 mainly interacts with archetypal PDI family members, whereas TXNDC5 mainly interacts with peroxiredoxin-4 (Prx4) [10, 17]. Hydrogen peroxide generated during Ero1α–PDI interaction can act on the catalytic oxidation reaction between Prx4 and TXNDC5 to accelerate protein folding [10]. In addition, TXNDC5 can interact with peroxidized Prx2 and participate in redox signal transduction [18].

The three Trx-like domains of TXNDC5 can act independently to introduce disulfide bonds rapidly but disorderly without efficient selectivity. The two Trx-like domains of PDI act synergistically, so it is not as fast as TXNDC5. However, PDI can selectively introduce disulfide bonds and correct for nonnatural disulfide bonds. Therefore, TXNDC5 and PDI may act at different stages of oxidative protein folding [10, 19]. In addition, TXNDC5 has a protective effect on endothelial cells in a hypoxic environment. Inhibiting the expression of TXNDC5 can lead to a decrease in the secretion of molecules that protect cells from hypoxia, such as adrenomedullin, endothelin-1, and CD105 [1]. Moreover, TXNDC5 is involved in TNFα-induced angiogenesis [20]. Knockout of TXNDC5 can prevent TNFα-induced phosphorylation of ERK1/2. Phosphorylated ERK is an important substance that promotes the expression of two important angiogenesis-related proteases, MMP-9 and cathepsin B [20]. TXNDC5 also has a molecular chaperone function that can promote the proper protein folding [21, 22]. ER degradation enhancing α -Mannosidase-like protein 3 (EDEM3) is a key enzyme that can remove mannose from *N*-polysaccharide and repair the abnormal folding protein. TXNDC5 can bind to EDEM3 and trigger its mannose pruning activity to correct misfolded proteins. [23].

### TXNDC5 and cancers

Currently, TXNDC5 is considered as a cancer-enhancing gene [24, 25]. It can protect cancer cells from oxidative stress and promote cancer growth, proliferation, migration and angiogenesis. Xu et al. found that TXNDC5 was a susceptibility gene for cervical cancer [26]. SNP analysis of the TXNDC5 gene found that two SNPs (rs1225944 and rs1225943) of TXNDC5 are related to the development of hepatocellular carcinoma (HCC) [27]. In addition, our research group found that the TXNDC5 gene increases the risk of cervical cancer, oesophageal cancer and liver cancer [21]. TXNDC5 is highly expressed in various cancer tissues. Therefore, many researchers have conducted extensive studies on its role in cancers.

### The mechanism of TXNDC5 promoting cancers

TXNDC5 can promote cancer development in many ways, and they are summarized in Table 2. Like other PDI family members, TXNDC5 can participate in the formation of disulfide bonds, promoting the correct folding of proteins in the ER and preventing endoplasmic reticulum stress. However, PDIs can protect cells under both normal and hypoxic conditions, whereas TXNDC5 has this function only under hypoxic conditions [1].

**Table 2:** Cancer-promoting mechanism of TXNDC5

Mechanism	Cancers	Regulator	TXNDC5 expression	Effect	Result	Ref
Regulate cell cycle	GC	–	Up	–	Increase percentage of cells in G2/M phase	[25]
	GC	–	Silence	–	Increase percentage of cells in 0/G1 phase	[25]
	CRPC	–	Up	–	Cause cell cycle disorders between G2/M and S phases	[29]
Protect cells from oxidative stress	CRC	Hif-1α	Up	Inhibit ROS/ER stress	Promote the reproduction and survival of cells under hypoxia	[28]
	CRPC	Hif-1α& miR-200b	Up	Interact with AR and enhance its transcriptional activity	promote the progression of CRPC	[29]
Signalling pathway	CC	–	Inhibition	Increase expression of SERPINF1 and TRAF1	Inhibit angioplasty, cell proliferation and migration	[26]
	LC	–	Up	Combine with Srx	Promote cell proliferation and metastasis	[30, 31]

*CRC* colorectal cancer, *CRPC* castration-resistant prostate cancer, *GC* gastric cancer, *CC* cervical cancer, *LC* lung cancer, *AR* androgen receptor, *Srx* sulfiredoxin

The expression of TXNDC5 is upregulated by hypoxia [21]. In vivo studies showed that hypoxia induces TXNDC5 expression by upregulating hypoxia inducible factor-1α (HIF-1α), thereby inhibiting hypoxia-induced ROS/ER stress signalling and promoting the reproduction and survival of colorectal cancer (CRC) cells [28]. However, TXNDC5 has no obvious effect on CRC cells under normoxia [28]. TXNDC5 can promote the progression of castration-resistant prostate cancer (CRPC) through androgen receptor (AR) signal transduction [29]. The specific mechanism is that androgen deprivation therapy (ADT) induces hypoxia in prostate cancer patients. In addition, hypoxia leads to increased expression of AR and TXNDC5. TXNDC5 can directly interact with AR and enhance its stability and transcriptional activity. Besides, TXNDC5 can make the Akt, ERK1/2, MET, and HER2 signalling pathways sensitive to hypoxia.

TXNDC5 can regulate the cell cycle. TXNDC5 can promote cell proliferation by regulating the cell cycle. Upregulating the expression of TXNDC5 in gastric cancer cells can significantly increase the percentage of cells in the G2/M phase, whereas downregulating can increase the percentage of cells in the 0/G1 phase [25]. In CRPC, TXNDC5 promotes cancer cell proliferation by causing cell cycle disorders between G2/M and S phases in vivo and in vitro [29].

TXNDC5 promotes cancer through distinct pathways. In vitro experiments have found that inhibiting the expression of TXNDC5 in cervical cancer cells leads to increased expression of SERPINF1 and TRAF1 [26]. SERPINF1 and TRAF1 can inhibit angiogenesis and metastasis and induce apoptosis. In lung cancer cells, TXNDC5 exerts a cancer-promoting effect by combining with sulfiredoxin (Srx) [30]. Srx can promote the proliferation, colony formation, and metastasis of lung cancer cells by activating mitogen-activated protein kinase cascades. TXNDC5 interacts with Srx to promote the retention of Srx in the ER, thereby protecting ER homeostasis of lung cancer cells and promoting the growth, proliferation, and invasion of cells [31]. In addition, TXNDC5 can interact with adiponectin receptor 1 (AdipoR1) in HeLa cells. Knocking out TXNDC5 can change the expression and distribution of AdipoR1 and adiponectin signals [32]. However, Duivenvoorden et al. found that TXNDC5 cannot establish a stable interaction with AdipoR1 in renal cell carcinoma (RCC) cells [33]. TXNDC5 may act through transient binding with AdipoR1. Alternatively, the difference in the reaction environment may affects the observed results of the experiment. In short, the relationship between AdipoR1 and TXNDC5 needs further research and exploration.

### Expression regulation of TXNDC5 in cancers

As mentioned above, the expression of TXNDC5 is up-regulated by hypoxia. In particular, the expression of TXNDC5 in non-small-cell lung cancer tissues is upregulated, but this regulation is not controlled by hypoxia [34]. Indeed, TXNDC5 expression is also regulated by a variety of molecules (Table 3). CIRC_0000517 is upregulated in HCC tissues and cells. It can promote TXNDC5 expression by downregulating miR-1296-5p, thereby promoting cell viability, proliferation, colony formation, and inhibiting apoptosis [35]. The combination of circRNA-104718 and miR-218-5p in HCC cells could promote the expression of TXNDC5 [36]. Human ether a-go-go-related gene 1 (HERG1) in esophageal squamous cell carcinoma (ESCC) can promote the expression of TXNDC5 and promote cancer progression by activating the PI3K/AKT pathway [37]. The nuclear receptor 4A1 (NR4A1) in pancreatic cancer cells can promote the expression of the TXNDC5 gene to resist ROS/ER stress and inhibit cell apoptosis [38]. NR4A1 has also been found to regulate the TXNDC5 gene in renal cell adenocarcinoma [39]. Inhibiting NR4A1 in rhabdomyosarcoma (RMS) cells can reduce the expression of TXNDC5, thereby reducing the production of ROS and IL-24 and inhibiting the proliferation, survival and migration of RMS cells [40].

**Table 3:** Expression regulation mechanism of TXNDC5 in cancer cells

Molecule	Cancers	Pathway	TXNDC5 expression	Effect	Ref
Circ_0000517	HCC	miR-1296-5p	Up	Promote proliferation and colony formation; inhibit apoptosis	[35]
HERG1	ESCC	PI3K/AKT	Up	Promote cancer progression	[37]
NR4A1	PC*/*RCC/RMS	–	Up	Inhibit apoptosis; anti-ROS/ER stress	[38–40]
Cetuximab	LSCC	Suppress TXNDC5 gene promoter	Down	Increase ERS stress-related apoptosis	[43]

HERG1 human ether a-go-go-related gene 1, *NR4A1* nuclear receptor 4A1, *HCC* hepatocellular carcinoma, *ESCC* Esophageal squamous cell carcinoma, *PC* pancreatic cancer, *RCC* renal cell carcinoma, *RMS* rhabdomyosarcoma, LSCC laryngeal squamous cell carcinoma

### The role of TXNDC5 in cancer diagnosis and treatment

TXNDC5 may be used as a diagnostic marker for a variety of cancers (Fig. 1d). Evidence obtained by immunohistochemical staining and Western blot showed that TXNDC5 expression was altered in a variety of cancers. Tan et al. found that the expression of TXNDC5 was positively correlated with the TNM stage of colorectal cancer [28]. TXNDC5 was upregulated in poorly differentiated HCC but did not change in highly differentiated HCC [41]. In addition, the expression of TXNDC5 was negatively correlated with the overall survival rate in RCC [42] and gastric cancer [24]. However, lung cancer patients with high TXNDC5 levels have a longer survival time [31]. These studies indicated that TXNDC5 may be used as a prognostic marker for cancers, and the same TXNDC5 expression status in different diseases may indicate different prognostic results.

Variant gastritis (VG) is a precancerous lesion of gastric cancer. Detection of differentially expressed proteins in VG and normal tissues revealed significant differences in TXNDC5 expression [25]. In addition, the content of TXNDC5 in relatively early stage lung cancer is higher than that in normal tissue [34]. The above results indicate that the change in TXNDC5 expression may have occurred in the cancers’ early stages. However, the sample size of the above studies was small, and the application prospects of TXNDC5 as a cancer marker need to be verified by large-scale experiments.

TXNDC5 can be used as a therapeutic target for cancers (Fig. 1e). Inhibiting TXNDC5 expression in cervical cancer [21], castration-resistant prostate cancer [29], RCC [34], gastric cancer [25], laryngeal squamous cell carcinoma (LSCC) [43], pancreatic cancer [38], and liver cancer [35] can promote cell apoptosis and inhibit cell proliferation and migration. The above results suggest that TXNDC5 can be used as a therapeutic target for cancers. Knockout of TXNDC5 can make clear cell renal cell carcinoma (ccRCC) cells allergic to chemotherapy drugs (such as camptothecin and 5-fluorouracil) and inhibit the growth, migration, and invasion of ccRCC cells [42]. Biochip research showed that after TXNDC5 knockdown, the expression of JAG1, STAT1, fibronectin, GLS, EFEMP1, PLK2, and EDN2 genes was downregulated, whereas caspase-7, CCDC80, CDK14, and dbicd2 genes were upregulated. These results indicate that TXNDC5 may play an important role in the carcinogenesis and development of ccRCC through complex signal crosstalk [42]. In addition, TXNDC5 may be a potential therapeutic target for LSCC. Cetuximab and cisplatin are commonly used chemotherapy drugs for LSCC. Peng et al. found that cetuximab could reduce TXNDC5 expression, thereby increasing the production of ROS and enhancing the endoplasmic reticulum stress-related apoptosis of LSCC cells [43].

### TXNDC5 and rheumatoid arthritis (RA)

In 2009, our research group found that TXNDC5 was specifically expressed in the tissues of RA patients compared with osteoarthritis and ankylosing spondylitis [44]. High expression of TXNDC5 in the blood and synovial fluid of RA patients can be detected in the early and continuous course of the disease [44]. The expression of TXNDC5 in tissues is positively correlated with the histological inflammatory score of osteoarthritis, chronic pyrophosphate arthropathy and RA [45]. In vivo experiments showed that TXNDC5-Tg mice are susceptible to collagen-induced arthritis (CIA); In vitro experiments showed that increased TXNDC5 expression in Rheumatoid arthritis synovial fibroblast-like cells (RASFs) could promote cell invasion, migration and secretion of TNF-α, IL-1α, IL-1β and IL-17. After treatment with anti-TXNDC5 siRNA, the above effects were weakened [46]. In addition, SNP analysis showed that 9 SNPs (rs9505298, rs41302895, rs1225936, rs1225938, rs372578, rs443861, rs408014, rs9392189, and rs2743992) were associated with RA [47].

**Fig. 2:**
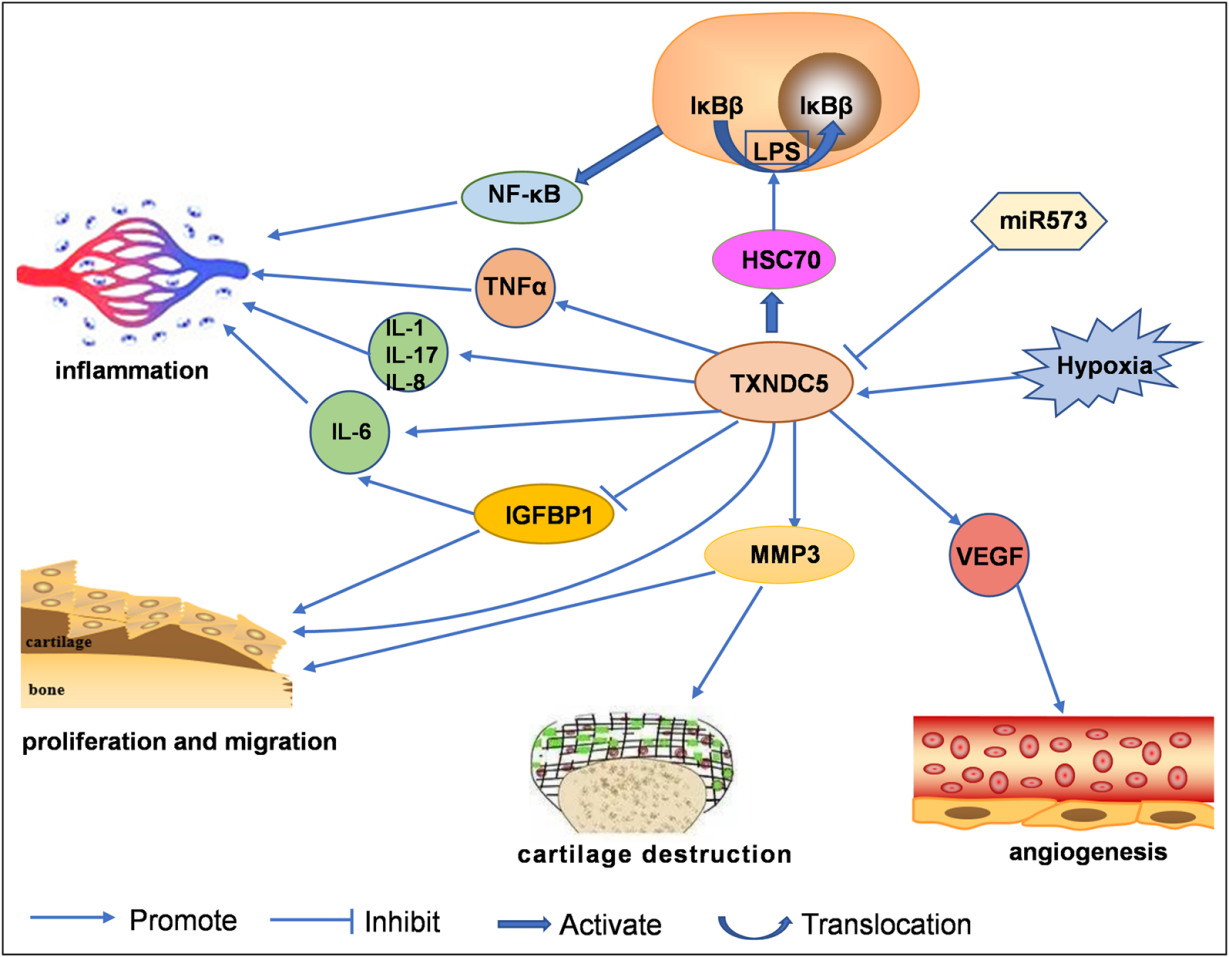
Role of TXNDC5 in rheumatoid arthritis (RA). TXNDC5 regulates RA progression, such as fibroblast proliferation and migration, inflammation, cartilage destruction and angiogenesis

In recent years, it has been found that TXNDC5 promotes the process of RA. TXNDC5 can protect synovial fibroblasts from the harmful effects of ER stress, thereby promoting the survival of cells [48]. In addition, TXNDC5 can promote angiogenesis and inflammation by inducing the secretion of vascular endothelial growth factor (VEGF), IL-6, and IL-8 [48]. TXNDC5 can inhibit the expression of insulin-like growth factor-binding protein-1 (IGFBP1). Insulin-like growth factor 1 (IGF-1) is a hormone with a structure similar to insulin, and it can bind to the IGF receptor. After binding, IGF exhibits tyrosine kinase activity. It can activate signalling pathways, such as phosphoinositide 3-kinase/protein kinase B (PI3K/AKT) and RAS/MAPK, thereby affecting cell proliferation, differentiation, survival and apoptosis [49]. TXNDC5 can also regulate NF-κB signalling by co-acting with the heat-shock-protein-70 (HSC70) [50]. In the absence of lipopolysaccharide (LPS), HSC70 can accelerate the degradation of IκBβ; In the presence of LPS, HSC70 can transfer more IκBβ into the nucleus [50]. TXNDC5 can regulate NF-κB signalling by retaining HSC70 in the cytoplasmic compartment. In addition, the expression of TXNDC5 can be regulated by miR573. Inhibiting miR-573 in RASFs can upregulate the expression of TXNDC5. TXNDC5 can enhance the migration of RASFs and cartilage destruction in RA by regulating the expression of matrix metalloproteinase-3 (MMP-3) [51]. The role and mechanism of TXNDC5 in RA mentioned above are shown in Table 4 and Fig. 2.

**Table 4:** Role and mechanism of TXNDC5 in RA

TXNDC5 expression	Pathway	Effect	Result	Ref
UP	–	Increase the secretion of TNF-α, IL-1α, IL-1β and IL-17	Promote cell invasion, migration and survival	[46]
UP	–	Induce the secretion of VEGF, IL-6, and IL-8	Promote inflammation and angiogenesis	[48]
UP	PI3K/AKT; RAS/MAPK	Inhibit the expression of IGFBP1	Affect cell proliferation, differentiation, survival and apoptosis	[49]
UP	co-acting with HSC70	Regulate NF-κB signalling	Regulate inflammatory response	[50]
UP	–	Regulate the expression of MMP-3	Enhance cartilage destruction and the migration of RASFs	[51]

*RASF* rheumatoid arthritis synovial fibroblast-like cell, *IGFBP1 *insulin-like growth factor-binding protein-1, *HSC70* heat-shock-protein-70, *MMP-3* matrix metalloproteinase-3

### TXNDC5 and other diseases

In addition to cancers and RA, the SNPs of TXNDC5 are also related to many other diseases. A study in Korean population found that three exon SNPs (rs1043784, rs7764128, and rs8643) in TXNDC5 are related to the pathogenesis/susceptibility of nonpigmented vitiligo [52]. The rs13873 SNP of the TXNDC5 gene and the rs1225934–rs13873 haplotype of the BMP6–TXNDC5 gene are related to sustained attention impairment in patients with schizophrenia [53]. In addition, a SNP related to TXNDC5 (rs13196892: TXNDC5 | MUTED) is one of the newly discovered menopausal/age-related SNPs in longevity populations [54]. These SNPs may cause disease by affecting the function of TXNDC5. In addition, TXNDC5 was found to be significantly increased in probands with potential new diseases, indicating that the gene may not easily tolerate copy damage [55]. Thus, the relationship between the SNPs of TXNDC5 and disease are worth further exploration.

An Arg345Trp (R345W) mutation in Fibrin-3 (F3) causes the rare, autosomal dominant macular dystrophy, Malattia Leventinese. John et al. found that TXNDC5 can interact with wild-type (WT) and R345W mutant F3 [56]. In vivo and in vitro experiments found that when interferon-stimulated gene 15 (Isg 15) was knocked out, the expression of TXNDC5 was reduced. It was suggested that Isg15 mediated its proviral role in hepatitis C virus replication through TXNDC5 [57]. Therefore, TXNDC5 is expected to serve as a target for gene or drug therapy in an effort to alter the fate of the above diseases.

TXNDC5 was increased in human and mouse atherosclerotic lesions. In addition, it could increase proteasome-mediated degradation of heat shock factor 1, leading to reduced heat shock protein 90 and accelerated eNOS (endothelial nitric oxide synthase) protein degradation. TXNDC5 deletion markedly increased eNOS protein and reduced atherosclerosis in ApoE −/− mice [58]. In addition, inhibiting the expression of TXNDC5 can reduce damage from isoproterenol to the heart, relieve fibrosis, and improve myocardial function [59]. In further research, it was found that in human cardiac fibroblasts (hCF), TGFβ1 can upregulate the expression of TXNDC5. TXNDC5 can promote ECM protein folding, thereby enhancing the JNK signaling pathway and promoting the activation and proliferation of hCF [59]. Similarly, the role of TXNDC5 in renal and pulmonary fibrosis via the TGF-β pathway has been revealed by related studies [60, 61]. In addition, it was found that inhibiting activity of IRE1α endoribonuclease can reduce the expression of TXNDC5 in activated fibroblasts and delay lung fibrosis induced by crystalline silica. [62].

Histone deacetylase inhibitor (HDACi) is an effective drug for cutaneous T-cell lymphoma (CTLC), but drug resistance often occurs in patients. The increased level of histone acetylation in drug-resistant CTLC patients is associated with increased expression of many genes, including TXNDC5 [63]. Therefore, the detection of TXNDC5 can be used to determine whether CTLC patients are resistant to HDACi. Analogously, the high expression of TXNDC5 in plasma cells of bortezomib-resistant refractory/relapsed multiple myeloma (RRMM) patients suggests that TXNDC5 may be a bortezomib resistance marker or a potential RRMM treatment target [64]. TXNDC5 expression was increased in the renal medulla samples of autosomal dominant tubulointerstitial kidney disease–UMOD model mice compared with normal mice [65]. In addition, the expression of TXNDC5 in peripheral blood mononuclear cells of the elderly is lower than that of young people, suggesting that expression changes in TXNDC5 may be related to the ageing of endothelial cells [66]. Kim et al. found that TXNDC5 expression decreased with age and may serve as a biomarker for skin ageing [67]. In addition, the TXNDC5 content in peripheral blood B cells can be used as a marker of the identification of seroconversion after COVID-19 vaccination [68]. Compared with wild-type mice, the expression of TXNDC5 were increased in the liver tissues of apoE-ko mice supplemented with squalene. The increase in TXNDC5 expression was related to liver fat content. This evidence suggests that TXNDC5 can be used as a target of squalene to combat hepatic steatosis [69]. The expression of TXNDC5 in lipid rafts of human umbilical vein endothelial cells treated with atorvastatin was upregulated, and the combination of TXNDC5 and Nox2 also increased. Atorvastatin may inhibit the activity of Nox2 by promoting the membrane translocation of TXNDC5 and the combination of TXNDC5 and Nox2, thereby exerting an antioxidant effect [70]. In addition, simvastatin enhances docetaxel-induced toxicity of human prostate cancer cells. In addition, TXNDC5 was found to be involved in this effect as the targets of simvastatin [71]. In summary, TXNDC5 plays an important role in various diseases, and it shows a potential in the diagnosis and treatment of diseases. The complex mechanisms and role of TXNDC5 in various diseases deserve further exploration.

## Conclusions

TXNDC5 is a member of the PDI family. It has three Trx-like domains that can promote correct protein folding. In addition, it can protect cells from oxidative stress, promote proliferation and inhibit apoptosis. High expression of TXNDC5 can promote the progression of cancer, RA, fibrosis, atherosclerosis and other diseases. Current studies indicated that TXNDC5 has a promising future in the diagnosis and treatment of diseases. Targeting TXNDC5 has also shown a significant role in the treatment of diseases. However, the limitations of detection methods and inclusion in larger databases make its role in disease difficult to fully understand. In the future, it is of great significance to further clarify the role and mechanism of TXNDC5 in different diseases. From laboratory to practical application, the study on TXNDC5 needs to be validated by large-scale clinical trials.

## Data Availability

Not applicable as this manuscript is a review article.

## Abbreviations

AdipoR1: Adiponectin receptor
ADT: Androgen deprivation therapy
AR: Androgen receptor
CIA: Collagen-induced arthritis
CRC: Colorectal cancer
CRPC: Castration-resistant prostate cancer
CTLC: Cutaneous T-cell lymphoma
EDEM3: ER degradation enhancing α-mannosidase-like protein 3
Ero1α: Endoplasmic reticulum oxidoreductase 1α
ESCC: Esophageal squamous cell carcinoma
HCC: Hepatocellular carcinoma
hCF: Human cardiac fibroblasts
HDACi: Histone deacetylase inhibitor
HERG1: Human ether a-go-go-related gene 1
HIF-1α: Hypoxia inducible factor-1α
HSC70: Heat-shock-protein-70
IGF-1: Insulin-like growth factor 1
IGFBP1: Insulin-like growth factor-binding protein-1
Isg 15: Interferon-stimulated gene 15
LPS: Lipopolysaccharide
LSCC: Laryngeal squamous cell carcinoma
MMP-3: Matrix metalloproteinase-3
NR4A1: Nuclear receptor 4A1
PDI: Protein disulfide isomerase
Prx4: Peroxiredoxin-4
RA: Rheumatoid arthritis
RASFs: Rheumatoid arthritis synovial fibroblast-like cells
RCC: Renal cell carcinoma
RMS: Rhabdomyosarcoma
RRMM: Refractory/relapsed multiple myeloma
SNP: Single nucleotide polymorphism
Srx: Sulfiredoxin
TXNDC5: Thioredoxin domain-containing protein 5
VEGF: Vascular endothelial growth factor
VG: Variant gastritis
